# Fractional Dynamics of HIV with Source Term for the Supply of New CD4^+^ T-Cells Depending on the Viral Load via Caputo–Fabrizio Derivative

**DOI:** 10.3390/molecules26061806

**Published:** 2021-03-23

**Authors:** Zahir Shah, Rashid Jan, Poom Kumam, Wejdan Deebani, Meshal Shutaywi

**Affiliations:** 1 Department of Mathematics, University of Lakki Marwat, Lakki Marwat 28420, Pakistan; zahir@ulm.edu.pk; 2 Center of Excellence in Theoretical and Computational Science (TaCS-CoE), Faculty of Science, King Mongkut’s, University of Technology Thonburi (KMUTT), 126 Pracha Uthit Rd., Bang Mod, Thung Khru, Bangkok 10140, Thailand; 3 School of Mathematics and Statistics, Xi’an Jiaotong University, Xi’an 710049, China; rashid_ash2000@yahoo.com; 4 KMUTT Fixed Point Research Laboratory, KMUTT-Fixed Point Theory and Applications Research Group, SCL 802 Fixed Point Laboratory, Department of Mathematics, Faculty of Science, King Mongkut’s, University of Technology Thonburi (KMUTT), 126 Pracha Uthit Rd., Bang Mod, Thung Khru, Bangkok 10140, Thailand; 5 Department of Medical Research, China Medical University Hospital, China Medical University, Taichung 40402, Taiwan; 6 Department of Mathematics, College of Science & Arts, King Abdulaziz University, P.O. Box 344, Rabigh 21911, Saudi Arabia; wdeebani@kau.edu.sa (W.D.); mshutaywi@kau.edu.sa (M.S.)

**Keywords:** human immunodeficiency virus, fractional dynamics, Caputo–Fabrizio derivative, numerical scheme, solutions pathway

## Abstract

Human immunodeficiency virus (HIV) is a life life-threatening and serious infection caused by a virus that attacks ${\mathrm{CD}4}^{+}$ T-cells, which fight against infections and make a person susceptible to other diseases. It is a global public health problem with no cure; therefore, it is highly important to study and understand the intricate phenomena of HIV. In this article, we focus on the numerical study of the path-tracking damped oscillatory behavior of a model for the HIV infection of ${\mathrm{CD}4}^{+}$ T-cells. We formulate fractional dynamics of HIV with a source term for the supply of new CD4^+^ T-cells depending on the viral load via the Caputo–Fabrizio derivative. In the formulation of fractional HIV dynamics, we replaced the constant source term for the supply of new ${\mathrm{CD}4}^{+}$ T-cells from the thymus with a variable source term depending on the concentration of the viral load, and introduced a term that describes the incidence of the HIV infection of ${\mathrm{CD}4}^{+}$ T-cells. We present a novel numerical scheme for fractional view analysis of the proposed model to highlight the solution pathway of HIV. We inspect the periodic and chaotic behavior of HIV for the given values of input factors using numerical simulations.

## 1. Introduction

Mathematical biology has a wide range of applications in genetics, environmental sciences, population dynamics, medical sciences, etc. The field may be expressed as biomathematics depending on whether dealing with mathematical aspects or biological aspects [1]. Several biological phenomena and processes can be formulating in the language of mathematics in the framework of ordinary differential equations, fractional differential equations, impulsive differential equations, stochastic differential equations, delay differential equations, etc. [2,3,4,5,6]. Different assumptions, laws, axioms governing these processes are used in the formulation of these mathematical models to show the intricate dynamics of biological phenomena. Presently, the most critical and dangerous global health issue is human immunodeficiency virus (HIV), and numerous mathematical models have been developed of the human immune system to highlight the overall picture of the infection that describes the infection of HIV and its interaction with the immune system. It is reported that HIV is the causative agent for obtaining acquired immune deficiency syndrome (AIDS), which damages the body’s ability to fight other infections. HIV is an incurable deadly disease, and millions of people have lost their lives from it. However, people are living long and healthy lives with HIV due to effective HIV treatment, care, diagnosis, and prevention. When HIV enters the body of a healthy individual, it quickly reproduces and damages the ${\mathrm{CD}4}^{+}$ T-cells, which affects the immune system. The symptoms in the primary stage of HIV are fever, night sweats, cough, weight loss, headache, diarrhea, rash, joint pain and muscle aches, swollen lymph glands, and sore throat. At this stage the load of viruses in the blood is high, and infection easily spreads in the body compared to other forthcoming stages. Furthermore, HIV infects via bodily fluids (blood, tears, urine, spit, etc.).

It is noted that ${\mathrm{CD}4}^{+}$ T-cells play a role in the human body and are the target cells of HIV; moreover, these ${\mathrm{CD}4}^{+}$ T-cells perform a key role in the adjustment of the immune order, so the declination of these cells can have widespread influence on causing damage to a working immune system. The level of concentration of these cells is used to determine the stage of HIV infection; therefore, it is valuable to determine its importance using a mathematical framework. Numerous mathematical models have been constructed to visualize the transmission phenomena of ${\mathrm{CD}4}^{+}$ T-cells with HIV. In the literature, Perelson et al. [7] developed a simple mathematical system that describes the dynamics of HIV infection in ${\mathrm{CD}4}^{+}$ T-cells. Furthermore, Perelson and Nelson [8] developed another model based on [7], which consists of four variables: the population of free HIV particles, latently infected, actively infected, and uninfected cells. The model demonstrates many characteristics observed clinically of AIDS, e.g., the reduction of ${\mathrm{CD}4}^{+}$ T-cells, and the low levels and long latency time of the free virus in a human host body. After that, Raun and Calshaw [9] reduced the model developed by Perelson et al. [8] into one consisting of three independent variables: HIV-free virus particles, and infected and uninfected T-cells. The study by Bushnaq et al. [10] investigated the stability existence of steady states of HIV/AIDS mode with fractional derivatives and explored the role of memory in the dynamics of HIV infection. The authors in [11,12,13,14] implemented different techniques and methods to show the path-tracking behavior of the dynamics of HIV. The main objective of the current study is to formulate the dynamics of HIV with a variable source term instead of a constant term for the supply of new ${\mathrm{CD}4}^{+}$ T-cells from the thymus. Furthermore, the mass action term kVT is introduced, which shows the infection caused by the virus V, interacting with uninfected ${\mathrm{CD}4}^{+}$ T-cells, causing the loss of free virus at the rate −kVT, where *k* is the rate of infection.

It is well known that fractional-order systems offer more reliable, deeper, more valuable and more accurate information about the dynamics of different diseases than classical integer-order systems [15,16,17,18]. Hereditary properties and a description of memory make them superior to integer-order models [19,20,21,22,23]; moreover, fractional-order models can easily explore and demonstrate the dynamics between two points. These new ideas have been effectively used in modeling real-world problems in physics, engineering, biology, economics, and several other areas [24,25,26,27,28,29,30,31]. In advance studies and in recent research, fractional calculus produced more accurate information about the dynamics of a system; in particular the dynamical behavior of infectious diseases can be more accurately highlighted through fractional calculus. In fractional calculus, Riemann–Liouville, Caputo, Hilfer and some other operators using the power-law kernel and encounter limitations in modeling natural phenomena. On the other hand, the Caputo–Fabrizio operator possess an exponential decay law kernel which produce more accurate results for natural phenomena. This new developed fractional operator contains stronger properties as compared to the other fractional operators. Therefore, we opt to investigate the dynamical behavior of HIV in the framework of the fractional derivative under the Caputo–Fabrizio operator to capture more accurate and more reliable information about the infection.

Motivated by the above accurate results of the fractional derivative, we opt to explore the fractional dynamics of HIV with a source term for the supply of new CD4^+^ T-cells depending on the viral load using the Caputo–Fabrizio derivative. We represent the basic results of fractional Caputo–Fabrizio (CF) derivative in the first section of the article. In the second section, a fractional model is formulated for HIV with a source term for the supply of new CD4^+^ T-cells depending on the viral load in the framework of Caputo–Fabrizio derivative. The proposed model of HIV infection is then investigated through mathematical skills. In the third section, a novel numerical scheme is presented to visualize the tracking path of HIV in the fractional framework. The dynamics of HIV infection is then investigated with the effect of fractional order numerically in section four. We observe that the input parameter ϑ has a significant effect on the dynamics of HIV infection and decreases the level of infection. In addition, we show the chaotic behavior of the system with variation of input parameters to conceptualize the chaotic pattern of HIV that justifies the suitability of the scheme. Our analysis predicts that fractional order can be used as a control parameter, and should be suggested to policymakers. Finally, concluding remarks and suggestions are presented in last section.

## 2. Structure of HIV Dynamics

To study HIV/AIDS, the most important requirement is to conceptualize the population dynamics of ${\mathrm{CD}4}^{+}$ T-cells. These cells are produced in the bone marrow, and premature cells are shifted to the thymus, where they receive extra differentiation and mature into immunocompetent ${\mathrm{CD}4}^{+}$ T-cells. In humans, the thymus reaches its maximum weight at the time of puberty and then slowly becomes complex. Thymus ejection from an adult has small effect, even though the thymus in adults is in a working state and its few lymphocytes perform as precursors of T-cells and immunocompetent T-cells. The focus of the presented model is on ${\mathrm{CD}4}^{+}$ T-cells. The succession of HIV infection can be calculated by the number of ${\mathrm{CD}4}^{+}$ T-cells, which tells us about initial symptoms. The interest of the present study is in observing the HIV infection model of ${\mathrm{CD}4}^{+}$ T-cells, which performs path-tracking damped oscillations. The mathematical model presented here depicts the behavior of the infected rate of ${\mathrm{CD}4}^{+}$ T-cells. The above assumptions of [13] lead us to the following system:

(1) $$\begin{array}{ll} \; & \frac{dT}{dt}=s-\mu_{T}T+rT\left(1-\frac{T+I}{T_{\max}}\right)-kVT,\\ \; & \frac{dI}{dt}=kVT-\mu_{I}I,\\ \; & \frac{dV}{dt}=N\mu_{I}I-\mu_{V}V, \end{array}$$

where the three terms T(t), I(t), and V(t) illustrate the quantity of healthy ${\mathrm{CD}4}^{+}$ T-cells in the blood, infected ${\mathrm{CD}4}^{+}$ T-cells, and free HIV particles, respectively. The values of parameters, constants, and initial values of state variables and their explanation of the above-mentioned model are given in Table 1. In the upcoming section, the model will be extended in a fractional framework.

## 3. Fractional Dynamics of HIV Infection

In this section, a variable source term s(V)=sexp(−κV) is incorporated into the above system of HIV infection instead of constant the terms used in [13]. The new source term expresses the generated number of new healthy T-cells from the thymus, which depends on the concentration of the viral load. The production of healthy T-cells is observed to decrease due to the increase in the size of viral load; therefore, the source term is considered to be variable rather than constant.

The numerical solution of the new model using standard parameters with $\kappa =1{\;\mathrm{mm}}^{-3},$ performs the key role in the depletion of healthy T-cells in such a way that their depletion is more gradual than in the previous model. Here, *s* indicates the concentration of new healthy T-cells that are generated from sources within the system, such as the thymus. Healthy T-cells can be increased rapidly through existing healthy T-cells, and κ is the constant. The infection model can be solved by a technique in which the system is extended by introducing a term kVT. Here, *k* behaves as a constant rate of infection. This kind of term has logical importance in such a way that the virus necessarily infects the healthy T-cells, so the chance of attacking healthy T-cells at a low rate can be assumed to be proportional to the product of *V* and *T*. For this reason, the infection takes place when the virus communicates with uninfected T-cells. First, the virus is induced in heathy T-cells, which makes these heathy T-cells irresistible at a rate of −kVT and then produces infected T-cells at a rate of kVT. During HIV infection, the rate of the virus never exceeds the number of healthy T-cells. Infection can also take place via the communication of cells with each other, i.e., by meeting infected T-cells with healthy T-cells. In the presented model, *V* and *T* depicts the virus rate and measure of healthy T-cell count in the blood, as, obviously, there is no compulsion for the infected virus to just stay in the blood. In fact, a very large number of healthy T-cells is in lymphoid tissues. However, according to the given data, the calculated number of viruses and healthy T-cells in the blood adequately indicates their volume in the whole body [8,32,33], just as one would assume for a system to be in equilibrium. This extended model gives more accurate and more realistic forecasting of the healthy T-cell depletion for a given time, and can be written in the following way in the fractional framework:

(2) $$\begin{array}{ll} \; & {}_{0}^{CF}D_{t}^{\vartheta }T=s\exp\left(-\kappa V\right)-\mu_{T}T+rT\left(1-\frac{T+I}{T_{\max}}\right)-kVT,\\ \; & {}_{0}^{CF}D_{t}^{\vartheta }I=kVT-\mu_{I}I,\\ \; & {}_{0}^{CF}D_{t}^{\vartheta }V=N\mu_{I}I-\mu_{V}V-kVT, \end{array}$$

where ${}_{0}^{CF}D_{t}^{\vartheta }$ indicates Caputo–Fabrizio fractional derivative of order ϑ. In the next part of the paper, we will discuss and represent the rudimentary knowledge of the Caputo–Fabrizio fractional derivative, which will be used for the analysis of our proposed HIV infection model.

### Fundamental Knowledge and Concept

Here, we will represent the basic definitions and results important for the analysis of HIV with the source term for the supply of new CD4^+^ T-cells depending on the viral load in a fractional Caputo–Fabrizio(CF) framework. The fundamental knowledge of Caputo–Fabrizio fractional derivative is given as follows:

**Definition** **1.** *Let us suppose $h\in H^{1}\left(a,b\right)$, where b is greater than a, then the CF derivative [31] of order ϑ is given by*

(3) $$\begin{array}{c} D_{t}^{\vartheta }\left(h\left(t\right)\right)=\frac{U(\vartheta )}{1-\vartheta }\int_{a}^{t}h^{\prime }\left(x\right)\exp\left[-\vartheta \frac{t-x}{1-\vartheta }\right]dx, \end{array}$$

*where ϑ∈[0,1] and U(τ) denotes normality with U(0)=U(1)=1 [31]. In this case, when $h\notin H^{1}\left(a,b\right)$, then the following fractional derivative is obtained*

(4) $$\begin{array}{c} D_{t}^{\vartheta }\left(h\left(t\right)\right)=\frac{\vartheta U(\vartheta )}{1-\vartheta }\int_{a}^{t}\left(h\left(t\right)-h\left(x\right)\right)\exp\left[-\vartheta \frac{t-x}{1-\vartheta }\right]dx. \end{array}$$

**Remark** **1.** *Let us take $\alpha =\frac{1-\vartheta }{\vartheta }\in \left[0,\infty \right)$ and $\vartheta =\frac{1}{1+\alpha }\in \left[0,1\right]$, then Equation (4) can be written in the following form*

(5) $$\begin{array}{c} D_{t}^{\vartheta }\left(h\left(t\right)\right)=\frac{M(\alpha )}{\alpha }\int_{a}^{t}h^{\prime }\left(x\right)e^{\left[-\frac{t-x}{\alpha }\right]}dx,\;M\left(0\right)=M\left(\infty \right)=1. \end{array}$$

*In addition to this,*

(6) $$\begin{array}{c} \lim_{\alpha ⟶0}\frac{1}{\alpha }\exp\left[-\frac{t-x}{\alpha }\right]=\delta \left(x-t\right). \end{array}$$

*In the next step, we will define the fractional integral, which was introduced in [34].*

**Definition** **2.** *Let h be a given function then the fractional integral is defined in the following manner*

(7) $$\begin{array}{c} I_{t}^{\vartheta }\left(h\left(t\right)\right)=\frac{2(1-\vartheta )}{\left(2-\vartheta )U(\vartheta \right)}h\left(t\right)+\frac{2\vartheta }{\left(2-\vartheta )U(\vartheta \right)}\int_{0}^{t}h\left(u\right)du,\;\;\;t\geq 0. \end{array}$$

*where 0<ϑ<1 and is the order of the above fractional integral.*

**Remark** **2.** *Further analysis of the above Definition 2 gives that*

(8) $$\begin{array}{c} \frac{2(1-\vartheta )}{\left(2-\vartheta )U(\vartheta \right)}+\frac{2\vartheta }{\left(2-\vartheta )U(\vartheta \right)}=1, \end{array}$$

*which gives $U\left(\vartheta \right)=\frac{2}{2-\vartheta }$, 0<ϑ<1. A new Caputo derivative of order ϑ was introduced by Nieto and Losada in [34] by using Equation (8) and is given by*

(9) $$\begin{array}{c} D_{t}^{\vartheta }\left(h\left(t\right)\right)=\frac{1}{1-\vartheta }\int_{0}^{t}h^{\prime }\left(x\right)\exp\left[\vartheta \frac{t-x}{1-\vartheta }\right]dx,\;\;\;0<\vartheta <1. \end{array}$$

## 4. Novel Numerical Scheme for Fractional Model

In this section, the main aim is to illustrate the dynamical behavior of the proposed fractional model (2) of HIV infection, numerically. Several numerical approaches have been introduced in the literature for Caputo–Fabrizio fractional systems to highlight their dynamics [35,36,37]. Here, we will use the scheme of [37] to represent the dynamics of our fractional system (2) of HIV infection, which is more reliable, easily implementable, and stable. The values of the parameter presented in Table 1 will be used for simulation purposes. First, we represent our proposed fractional model of HIV infection in Volterra type and then apply the idea of a fundamental theorem of integration for further simplification. To obtain the numerical scheme, the first equation of our proposed model of HIV infection implies:

(10) $$z_{1}\left(t\right)-z_{1}\left(0\right)=\frac{1-\vartheta }{U(\vartheta )}H_{1}\left(t,z_{1}\right)+\frac{\vartheta }{U(\vartheta )}\int_{0}^{t}H_{1}\left(\omega ,z_{1}\right)d\omega .$$

Next, we take the time $t=t_{m+1},m=0,1,\cdots ,$ and get the following

(11) $$z_{1}\left(t_{m+1}\right)-z_{1}\left(0\right)=\frac{1-\vartheta }{U(\vartheta )}H_{1}\left(t_{m},z_{1}\left(t_{m}\right)\right)+\frac{\vartheta }{U(\vartheta )}\int_{0}^{t_{m+1}}H_{1}\left(t,z_{1}\right)dt.$$

and

(12) $$z_{1}\left(t_{m}\right)-z_{1}\left(0\right)=\frac{1-\vartheta }{U(\vartheta )}H_{1}\left(t_{m-1},z_{1}\left(t_{m-1}\right)\right)+\frac{\vartheta }{U(\vartheta )}\int_{0}^{t_{m}}H_{1}\left(t,z_{1}\right)dt.$$

Here, the difference in the successive terms of the system are obtained as follows:

(13) $$z_{1_{m+1}}-z_{1_{m}}=\frac{1-\vartheta }{U(\vartheta )}\left(H_{1}\left(t_{m},z_{1_{m}}\right)-H_{1}\left(t_{m-1},z_{1_{m-1}}\right)\right)+\frac{\vartheta }{U(\vartheta )}\int_{m}^{t_{m+1}}H_{1}\left(t,z_{1}\right)dt.$$

Furthermore, we approximate the above-mentioned function $H_{1}\left(t,z_{1}\right)$ in the time interval $\left[t_{k},t_{k+1}\right]$ with the help of interpolation polynomial and get

(14) $$P_{k}\left(t\right)≅\frac{H_{1}\left(t_{k},z_{k}\right)}{h}\left(t-t_{k-1}\right)-\frac{H_{1}\left(t_{k-1},z_{k-1}\right)}{h}\left(t-t_{k}\right),$$

in which *h* is the time spent and $h=t_{m}-t_{m-1}$. The expression of $P_{k}\left(t\right)$ is used to determine the value of the following integral

(15) $$\begin{array}{ll} \int_{m}^{t_{m+1}}H_{1}\left(t,z_{1}\right)dt & =\int_{m}^{t_{m+1}}\left(\frac{H_{1}\left(t_{m},z_{1_{m}}\right)}{h}\left(t-t_{m-1}\right)-\frac{H_{1}\left(t_{m-1},z_{1_{m-1}}\right)}{h}\left(t-t_{m}\right)\right)dt,\\ \; & =\frac{3h}{2}H_{1}\left(t_{m},z_{1_{m}}\right)-\frac{h}{2}H_{1}\left(t_{m-1},z_{1_{m-1}}\right). \end{array}$$

Here, substituting the value of (15) in Equation (13), we get the below

(16) $$\begin{array}{ll} z_{1_{m+1}} & =z_{1_{m}}+\left(\frac{1-\vartheta }{U(\vartheta )}+\frac{3\vartheta h}{2U(\vartheta )}\right)H_{1}\left(t_{m},z_{1_{m}}\right)\\ \; & -\left(\frac{1-\vartheta }{U(\vartheta )}+\frac{\vartheta h}{2U(\vartheta )}\right)H_{1}\left(t_{m-1},z_{1_{m-1}}\right), \end{array}$$

which is the required scheme for first equation of model (2) of HIV infection. Following the same procedure, we can determine the required scheme for the second and third equations of the proposed model (2) of HIV infection given by

(17) $$\begin{array}{ll} z_{2_{m+1}} & =z_{2_{m}}+\left(\frac{1-\vartheta }{U(\vartheta )}+\frac{3\vartheta h}{2U(\vartheta )}\right)H_{2}\left(t_{m},z_{2_{m}}\right)\\ \; & -\left(\frac{1-\vartheta }{U(\vartheta )}+\frac{\vartheta h}{2U(\vartheta )}\right)H_{2}\left(t_{m-1},z_{2_{m-1}}\right), \end{array}$$

and

(18) $$\begin{array}{ll} z_{3_{m+1}} & =z_{3_{m}}+\left(\frac{1-\vartheta }{U(\vartheta )}+\frac{3\vartheta h}{2U(\vartheta )}\right)H_{3}\left(t_{m},z_{3_{m}}\right)\\ \; & -\left(\frac{1-\vartheta }{U(\vartheta )}+\frac{\vartheta h}{2U(\vartheta )}\right)H_{3}\left(t_{m-1},z_{3_{m-1}}\right). \end{array}$$

**Figure 1:**
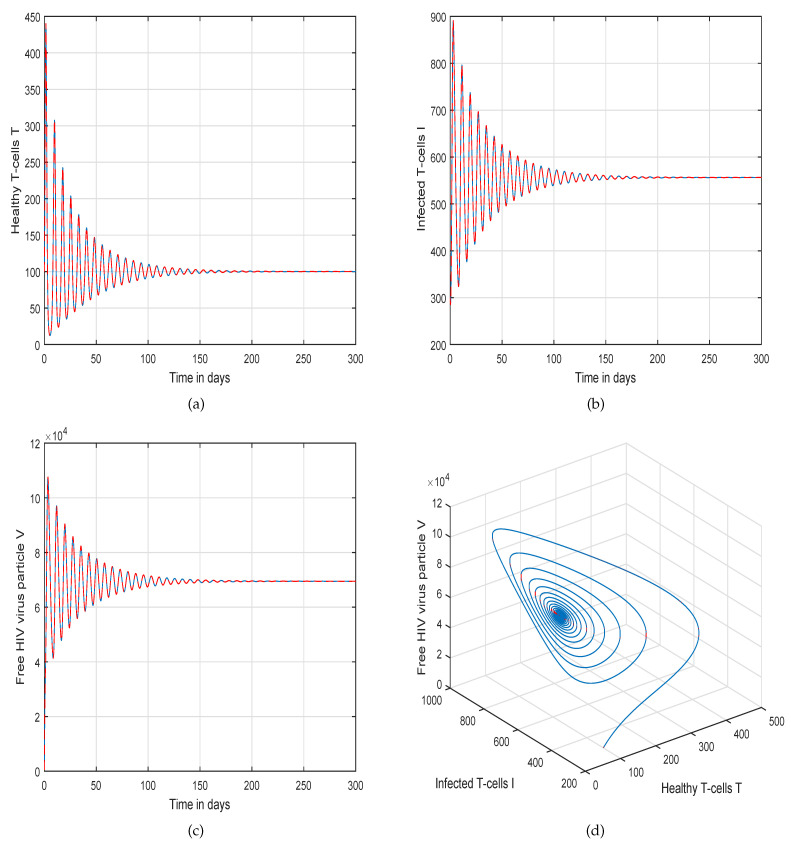
Illustration of the solution pathway of (**a**) healthy CD4^+^ T-cells, (**b**) infected CD4^+^ T-cells, (**c**) free HIV virus particles and (**d**) chaotic behavior of the proposed fractional model (2) of HIV infection with fractional-order *ϑ* = 1.

**Figure 2:**
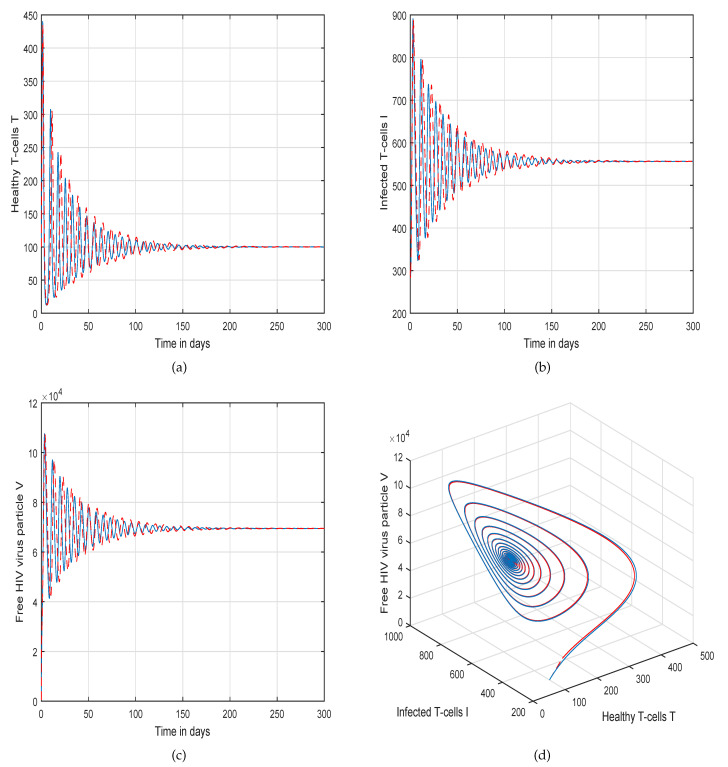
Illustration of the solution pathway of (**a**) healthy CD4^+^ T-cells, (**b**) infected CD4^+^ T-cells, (**c**) free HIV virus particles and (**d**) chaotic behavior of the proposed fractional model (2) of HIV infection with fractional-order *ϑ* = 0.85.

**Figure 3:**
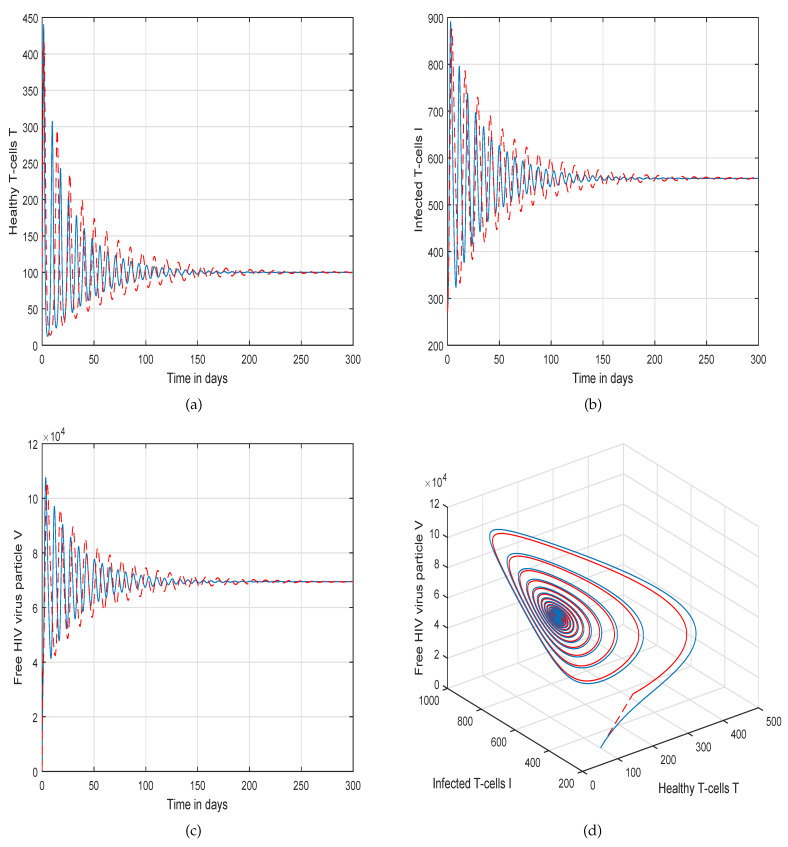
Illustration of the solution pathway of (**a**) healthy CD4^+^ T-cells, (**b**) infected CD4^+^ T-cells, (**c**) free HIV virus particles and (**d**) chaotic behavior of the proposed fractional model (2) of HIV infection with fractional-order *ϑ* = 0.65.

**Figure 4:**
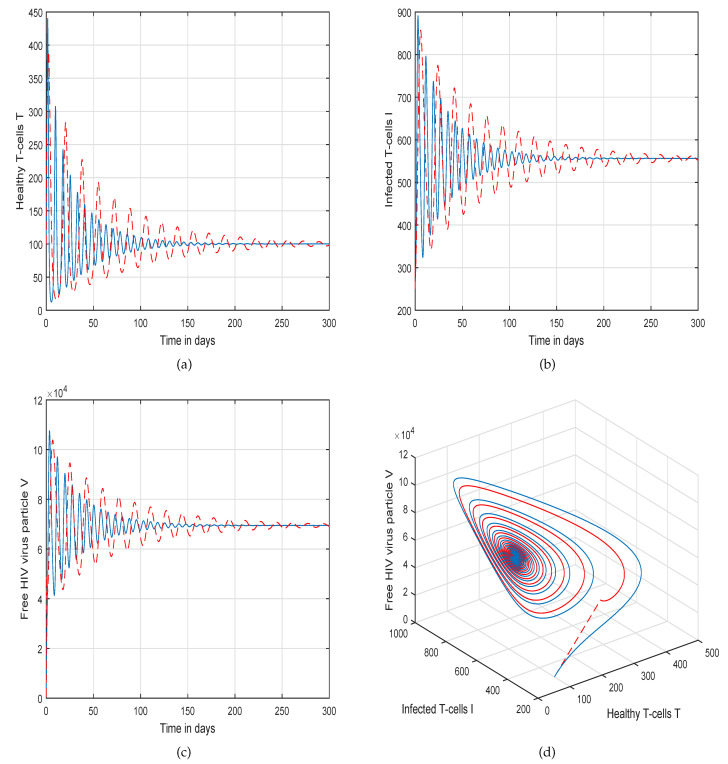
Illustration of the solution pathway of (**a**) healthy CD4^+^ T-cells, (**b**) infected CD4^+^ T-cells, (**c**) free HIV virus particles and (**d**) chaotic behavior of the proposed fractional model (2) of HIV infection with fractional-order *ϑ* = 0.45.

**Figure 5:**
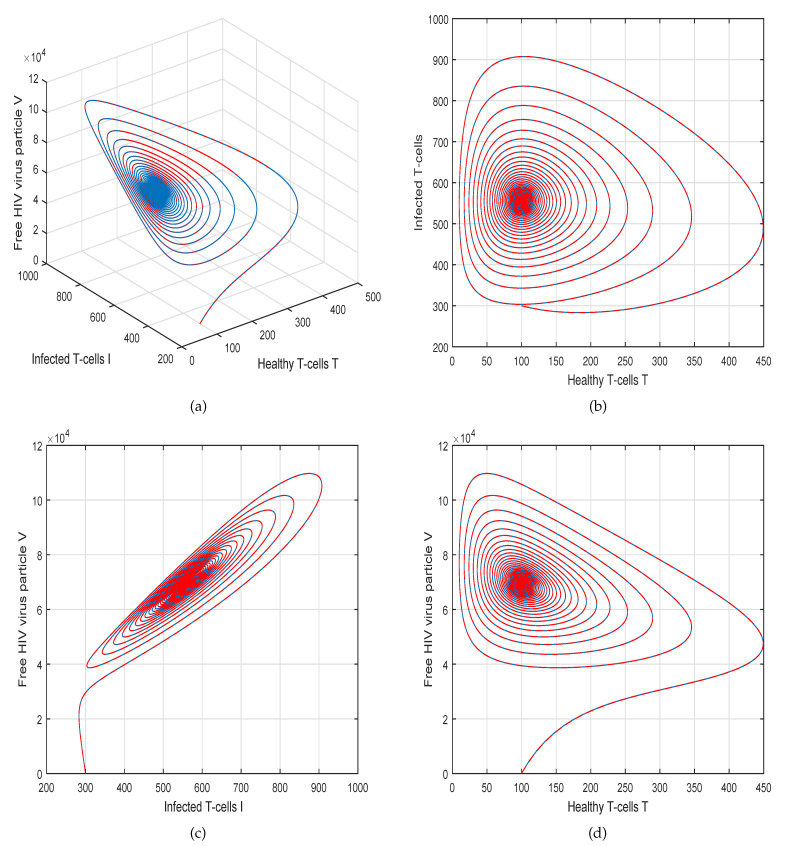
Illustration of the (**a**) chaotic behavior, (**b**) phase portrait of healthy CD4^+^ T-cells verses infected CD4^+^ T-cells, (**c**) phase portrait of infected CD4^+^ T-cells verses free HIV virus particles and (**d**) phase portrait of healthy CD4^+^ T-cells verses free HIV virus particles of the fractional model (2) of HIV infection with fractional-order *ϑ* = 1.

**Figure 6:**
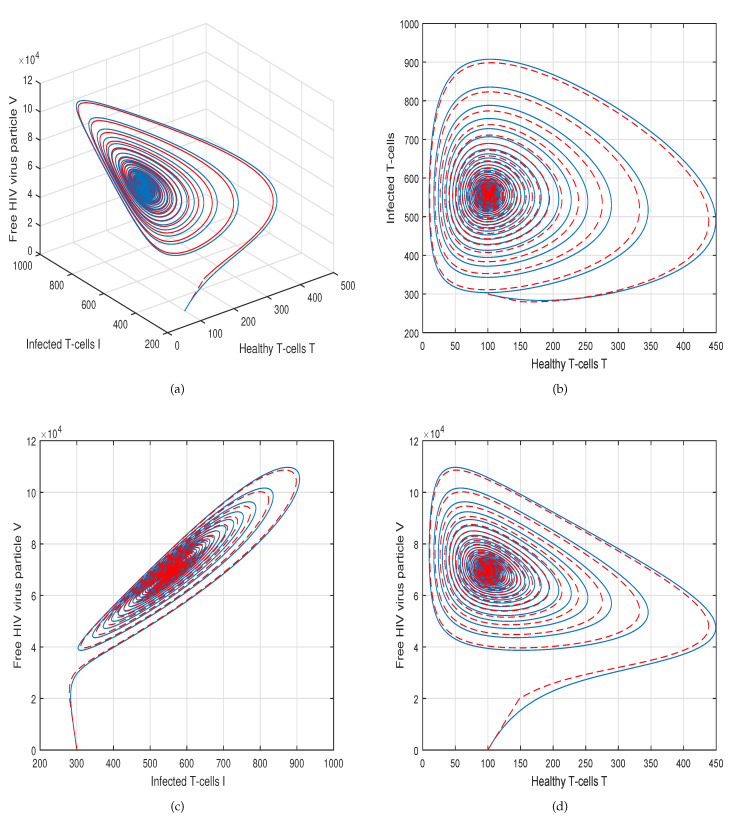
Illustration of the (**a**) chaotic behavior, (**b**) phase portrait of healthy CD4^+^ T-cells verses infected CD4^+^ T-cells, (**c**) phase portrait of infected CD4^+^ T-cells verses free HIV virus particles and (**d**) phase portrait of healthy CD4^+^ T-cells verses free HIV virus particles of the fractional model (2) of HIV infection with fractional-order *ϑ* = 0.8.

**Figure 7:**
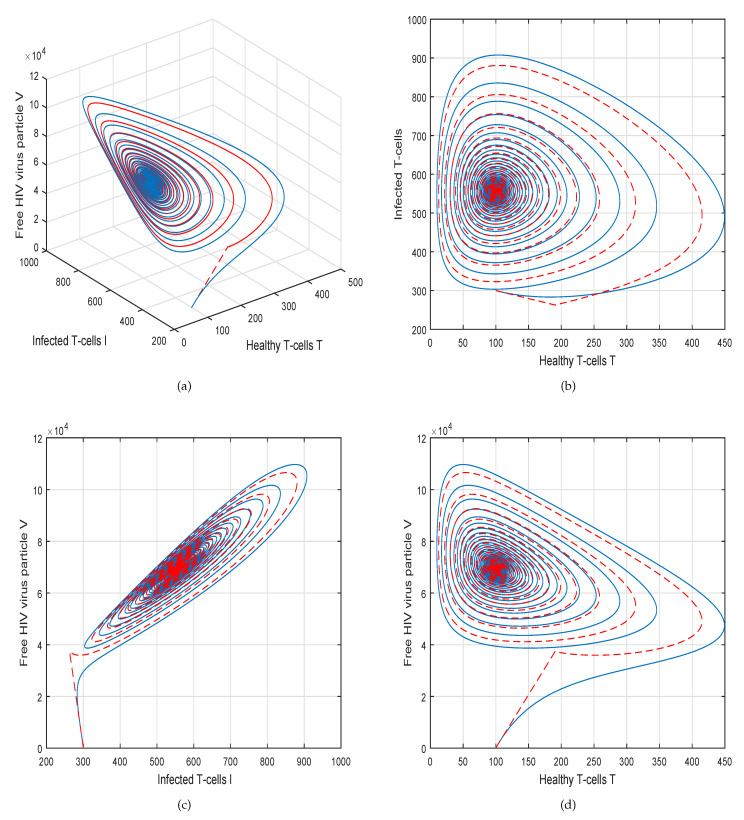
Illustration of the (**a**) chaotic behavior, (**b**) phase portrait of healthy CD4^+^ T-cells verses infected CD4^+^ T-cells, (**c**) phase portrait of infected CD4^+^ T-cells verses free HIV virus particles and (**d**) phase portrait of healthy CD4^+^ T-cells verses free HIV virus particles of the fractional model (2) of HIV infection with fractional-order *ϑ* = 0.6.

**Figure 8:**
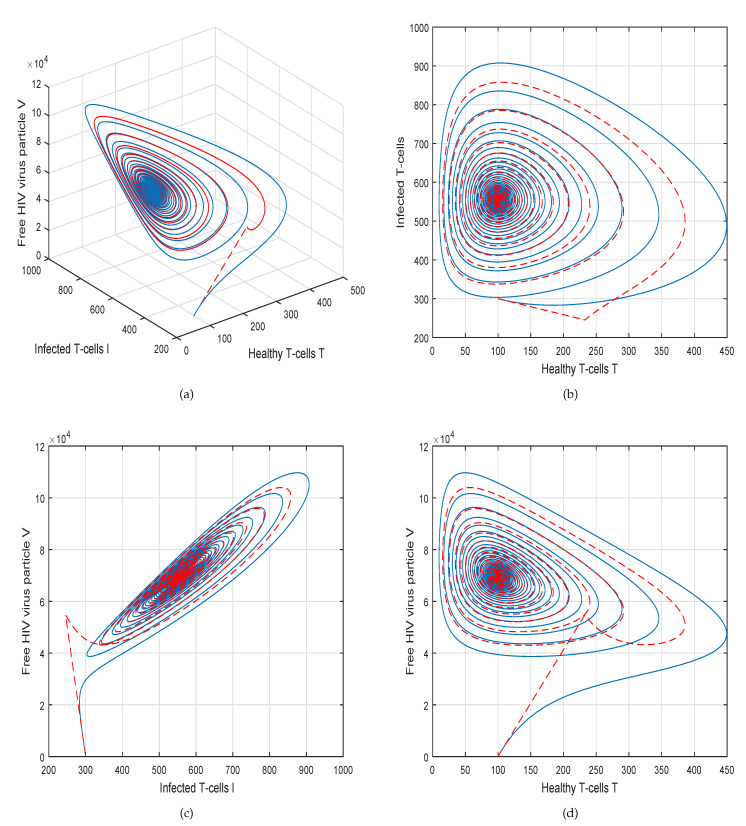
Illustration of the (**a**) chaotic behavior, (**b**) phase portrait of healthy CD4^+^ T-cells verses infected CD4^+^ T-cells, (**c**) phase portrait of infected CD4^+^ T-cells verses free HIV virus particles and (**d**) phase portrait of healthy CD4^+^ T-cells verses free HIV virus particles of the fractional model (2) of HIV infection with fractional-order *ϑ* = 0.4.

This method is a two-step Adams–Bashforth scheme for a Caputo–Fabrizio operator, which takes into account the nonlinearity of the kernel including exponential decay law for the Caputo–Fabrizio operator. In [37], the stability and convergence of the proposed scheme are presented in detail. We perform several simulations to understand the intricate dynamics of HIV, with the source term for the supply of new CD4^+^ T-cells depending on the viral load in the fractional framework. The values of input parameters and initial values of state variables given in Table 1 are used for simulation purposes. In Figure 1, Figure 2, Figure 3 and Figure 4, we demonstrate the dynamical behavior of healthy CD4^+^ T-cells, infected CD4^+^ T-cells, and HIV-free virus by varying the fractional-order ϑ, i.e., ϑ=1.0,0.85,0.65,0.45 to observe the influence of fractional-order ϑ on the dynamics of HIV infection. We noticed that the index of memory or fractional order is an effective input parameter and can play an important role in visualizing a more accurate picture of the system; furthermore, it can be used as a preventive parameter for HIV infection. In Figure 5, Figure 6, Figure 7 and Figure 8, we demonstrate the chaotic behavior of our fractional model (2) of HIV infection with different values of fractional-order ϑ. The chaotic behavior of a system is very important in many scientific and engineering applications. It is well known that there is a strong tendency towards the formulation and visualization of chaotic behaviors of a system. The chaotic patterns justify the suitability and applicability of the proposed numerical scheme, and can be further applied to novel chaotic systems. From our simulations, we observe that the input parameter ϑ highly contributes to and can be used as an effective parameter for preventive measures.

In our analysis, we mainly focused on the solution pathway and chaotic behavior of the HIV infection. In addition to this, we showed the influence of fractional parameter ϑ on the system to observe whether the fractional order can be used as a preventive measure or not. It should be noted in simulations that the proposed numerical scheme is less time-consuming and a more easily implementable scheme. However, the investigation of effectiveness of this scheme such as stability, convergence, consistency, accuracy, and computational cost are left for future research.

## 5. Conclusions

In this article, we formulated the fractional dynamics of HIV with a source term for the supply of new CD4^+^ T-cells depending on the viral load via a Caputo–Fabrizio derivative. In our model, we incorporated a variable source term for the supply of new ${\mathrm{CD}4}^{+}$ T-cells from the thymus depending on the concentration of the viral load. A rudimentary knowledge of fractional calculus in a Caputo–Fabrizio framework has been presented for the analysis of the model. We presented a novel numerical scheme for the solution of the Caputo–Fabrizio fractional model of HIV infection. The dynamics of HIV infection are demonstrated numerically with different values of fractional-order ϑ. The influence of fractional order on the chaotic behavior of our proposed model is illustrated. It is predicted that the index of memory ϑ has a positive effect on the system and can be used as a control parameter. Based on our analysis, we suggest that policymakers should not ignore the role of fractional order in the control of infections. In future research, we will compare the proposed numerical method with other existing numerical techniques and the dynamics of HIV infection will be presented with a time delay visualizing the effect of time delay on HIV infection.

## Figures and Tables

**Table 1:** List of parameters and state variables with corresponding values used in the fractional model of HIV infection.

Symbols	Interpretation of Parameters and State Variables	Values
*k*	Rate constant for healthy T-cells which become infected by free virus	$2.4\times 10^{-5}\;{\mathrm{days}}^{-1}$
$T_{0}$	Population of healthy T-cells	Assumed
$I_{0}$	Population of infected T-cells	Assumed
*s*	The supply rate of healthy T-cells from precursors	$0.1\;{\mathrm{mm}}^{-3}$
*r*	Growth rate of the healthy T-cells population	$3\;{\mathrm{day}}^{-1}$
$\mu_{T}$	Death rate of healthy T-cells	$0.02\;{\mathrm{day}}^{-1}$
$\mu_{I}$	Death rate of latently infected T-cells	$0.3\;{\mathrm{day}}^{-1}$
$\mu_{V}$	Death rate of free virus of HIV infection	$2.4\;{\mathrm{day}}^{-1}$
*N*	Number of virus produced by infected T-cells	Assumed
$T_{\max}$	Maximum population level of healthy T-cells	$1500\;{\mathrm{mm}}^{-3}$
$V_{0}$	Population of free HIV virus	Assumed
